# Alterations in mitochondria isolated from peripheral blood mononuclear cells and tumors of patients with epithelial ovarian cancers

**DOI:** 10.1038/s41598-023-51009-z

**Published:** 2024-01-02

**Authors:** Kittipat Charoenkwan, Nattayaporn Apaijai, Sirawit Sriwichaiin, Nipon Chattipakorn, Siriporn C. Chattipakorn

**Affiliations:** 1 Division of Gynecologic Oncology, Department of Obstetrics and Gynecology, Faculty of Medicine, Chiang Mai University, Chiang Mai, 50200 Thailand; 2 Neurophysiology Unit, Cardiac Electrophysiology Research and Training Center, Faculty of Medicine, Chiang Mai University, Chiang Mai, 50200 Thailand; 3 Center of Excellence in Cardiac Electrophysiology Research, Chiang Mai University, Chiang Mai, 50200 Thailand; 4 Cardiac Electrophysiology Unit, Department of Physiology, Faculty of Medicine, Chiang Mai University, Chiang Mai, 50200 Thailand; 5 Department of Oral Biology and Diagnostic Sciences, Faculty of Dentistry, Chiang Mai University, Chiang Mai, 50200 Thailand

**Keywords:** Medical research, Molecular medicine, Oncology

## Abstract

Metabolic alterations play an essential role in ovarian carcinogenesis. The flexibility of mitochondrial functions facilitates cellular adaptation to the tough environment associated with carcinogenesis. An understanding of the differences in mitochondrial functions in normal ovaries and cancers could provide a basis for further exploration of future mitochondria-based screening, diagnosis, prognostic prediction, and targeted therapy for epithelial ovarian cancers. The main objective of this study was to assess mitochondrial function profiles measured from PBMCs and ovarian tissues of epithelial ovarian cancers in comparison with normal ovaries. A total of 36 patients were recruited for the study, all of whom underwent primary surgical treatment for malignant epithelial ovarian neoplasm. Of these, 20 patients were in the early stage and 16 patients were in the advanced stage. Additionally, 21 patients who had pelvic surgery for benign gynecologic conditions, with normal ovaries incidentally removed, were recruited as controls. At the time of surgery, a blood sample was collected from each participant for PBMC isolation, and ovarian tissue was retained for molecular studies. These studies included the examination of oxidative stress, mitochondrial mass, mitochondrial respiration, mitochondrial reactive oxygen species (ROS), mitochondrial membrane potential (MMP) changes, and mitochondrial swelling. Clinical and histopathological data were also collected and compared between different stages of epithelial ovarian cancers: early-stage (group 1), advanced-stage (group 2), and normal ovaries (group 3). The levels of cellular oxidative stress, mitochondrial mass, and mitochondrial biogenesis in the peripheral blood mononuclear cells (PBMCs) of participants with ovarian cancer were significantly lower than those of the control group. However, the mitochondrial respiratory parameters measured from the PBMCs were similar across all three groups. Furthermore, mitochondrial membrane depolarization and mitochondrial swelling were observed in ovarian tissues of both early-stage and advanced-stage cancer groups. We demonstrated the dynamic nature of mitochondrial ROS production, biogenesis, and respiratory function in response to epithelial ovarian carcinogenesis. The flexibility of mitochondrial functions under diverse conditions may make it a challenging therapeutic target for ovarian cancer.

## Introduction

Ovarian cancer is one of the leading causes of cancer death in many parts of the world. It has been estimated that the lifetime risk of developing ovarian cancer is 1 in 70, and the risk of death from ovarian cancer is 1 in 100 in developed countries^1^. Generally, there are four histologic categories of ovarian cancers: surface epithelial-stromal tumor (most common), germ cell tumor, sex cord-stromal tumor, and secondary metastatic tumor. The surface epithelial-stromal tumors or “epithelial tumors” mainly involve women in their 50–60 s and present a major health threat to this population of women^2^.

It has been reported that metabolic alterations/remodeling plays an essential role in the development and progression of cancer, and its resistance to chemotherapy^3^. The vital metabolic derangements include aerobic glycolysis and macromolecular synthesis, resulting in anti-apoptotic effects on cancer cells^3^. Data from recent reports demonstrated that metabolic derangement occurred in ovarian cancers^4–6^. Apart from its conventional tumor suppressive role, the tumor protein p53 also plays a regulator role in cellular metabolic pathways including glycolysis, amino acid metabolism, lipid and lipoprotein metabolism, and oxidative phosphorylation^7–10^. In high-grade serous ovarian carcinoma, it has been reported that mutant p53 promotes lipid anabolism through increased expression of primary enzymes involving in the biosynthesis of fatty acids and cholesterol and inhibition of fatty acid oxidation. This process accelerates cancer growth and progression^11^. Similarly, serine-threonine kinase (Akt), a key glycolysis regulator, was found to be overexpressed in ovarian cancer, which leads to increased stability of the outer mitochondrial membrane, thus preventing apoptotic cell death^12^. These findings support the intimate relationship between energy metabolism and ovarian cancer carcinogenesis.

Mitochondria are intracellular organelles with a vital function in energy production by generating adenosine triphosphate (ATP) through oxidative phosphorylation. Apart from energy production, mitochondria have many other physiological functions that facilitate cellular adaptation to the tough environments associated with carcinogenesis such as hypoxia, nutritional starvation, and chemotherapeutic/targeted therapy. The flexibility of mitochondrial functions occur by balancing their biogenesis and mitophagy, fission and fusion dynamics, apoptosis, oxidative stress, metabolic signaling, and mitochondrial DNA mutations^13,14^. Removing these flexibilities afforded to the cancer cells by their mitochondria could play an important role in improving cancer treatment^15^. However, these functions vary in accordance with genetic, environmental, and tumor-site specific factors^13–15^. An in-depth understanding of the differences in mitochondrial functions in normal ovaries and cancers could provide a basis for further exploration of future mitochondria-based screening, diagnosis, prognostic prediction, and targeted therapy for epithelial ovarian cancers. The consequences of cellular stress from peripheral blood mononuclear cells (PBMCs) can serve as biomarkers for the progression of several diseases, such as neurodegeneration, diabetes, cardiovascular and cancers^16–20^. However, the correlations between mitochondrial functions from PBMCs, ovarian tissues, and other known major prognostic tumor biomarkers, including CA125 and CA19-9, have not been investigated. That information would offer valuable translational insight regarding the role of mitochondrial functions as potential prognostic markers for epithelial ovarian cancer.

The main objective of this study was to assess the profiles of mitochondrial functions measured from both PBMCs, including oxidative stress, mitochondrial mass, and mitochondrial respiration, and ovarian tissues, such as mitochondrial reactive oxygen species (ROS), mitochondrial membrane potential (MMP) changes, and mitochondrial swelling, of different stages of epithelial ovarian cancers in comparison with normal ovaries. We also investigated the correlation between the specified mitochondrial functions from PBMCs, ovarian tissues, and tumor biomarkers.

## Materials and methods

### Study population

Thirty-six patients with malignant epithelial ovarian neoplasm, including 20 patients in early-stage and 16 patients in advanced-stage, who had undergone primary surgical treatment at our institution were recruited. In addition, twenty-one patients who had pelvic surgery for benign gynecologic conditions and had normal ovaries incidentally removed along with the uterus were recruited as a control group. Exclusion criteria included histologically confirmed borderline ovarian tumor, secondary metastatic ovarian carcinoma originating from other organs and ovarian cancer patients who had received chemotherapy. In this study, patients who received chemotherapy were excluded, and the data analysis was conducted only on patients who did not receive chemotherapy. Further exclusions included patients whose remaining ovarian specimen was considered by the surgeons or the pathologists as inadequate for pathological evaluation if ovarian tissue sample were taken for this project.

### Participant recruitment

**Figure 1:**
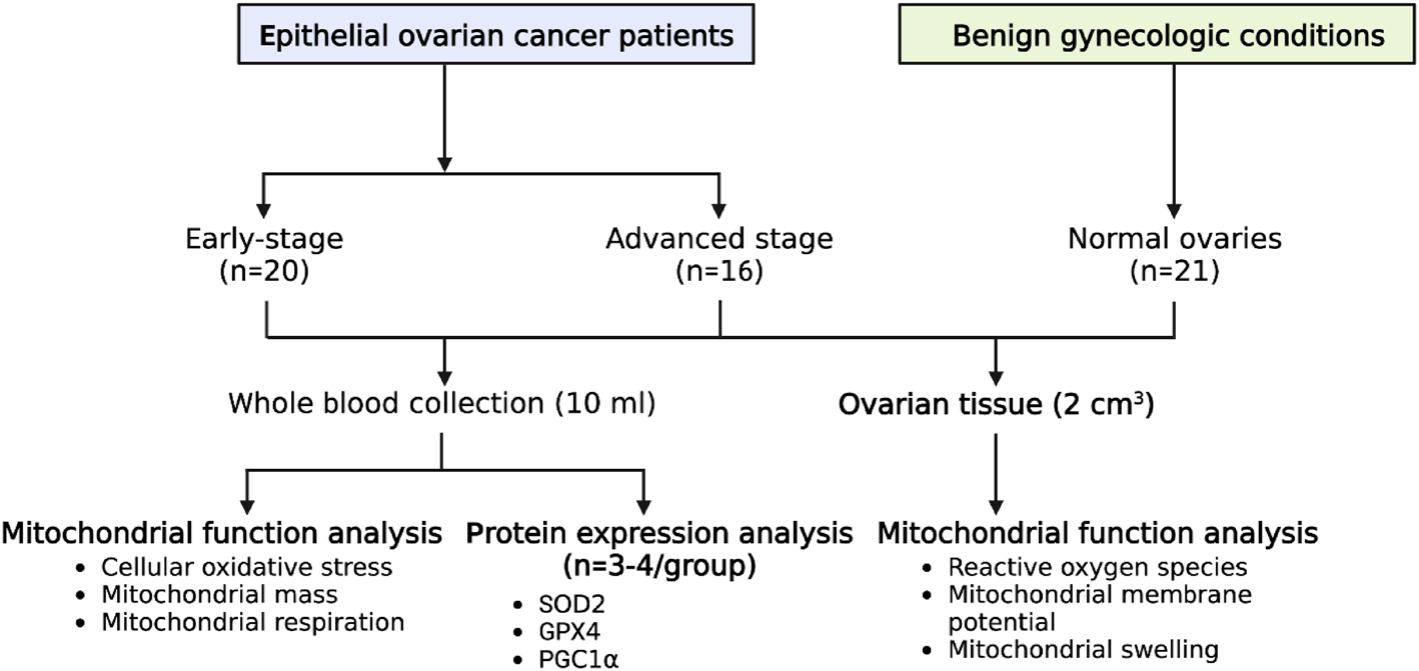
The protocol of the study.

The study commenced after approval by the Faculty of Medicine, Chiang Mai University Research Ethic Committee (approval number 075/2018). All methods were also performed in accordance with the relevant guidelines and regulations by the Faculty of Medicine, Chiang Mai University Research Ethic Committee. All eligible patients were invited to participate. Informed consent was obtained from each participant. The experimental protocol is shown in (Fig. 1).

### Mitochondrial function assessment

#### Ovarian tissue retrieval

For the patients included in this study, preoperative preparation and investigations were conducted as per routine standard practice. Approximately 2 cm^3^ of removed ovarian tissues were collected for mitochondrial function assessment. Clinical and detailed histopathological data of these patients were prospectively collected. Following surgery, all participants were treated, and followed as per clinical indications according to the standard protocol.

#### Mitochondrial isolation and mitochondrial function measurement in the ovarian tissues

Mitochondria were isolated from the fresh ovarian tissue. Tissue was homogenized in an isolation buffer, the homogenate was then subjected to differential centrifugation as previously described^21^. Mitochondrial protein at a concentration of 0.4 mg/ml was used for mitochondrial function analysis. Isolated mitochondria were incubated with 2 μM DCFH-DA dye (Sigma-Aldrich, USA) for 20 min at room temperature to determine mitochondrial oxidative stress levels. The fluorescence intensity of DCF was measured using a fluorescent microplate reader (BioTek, USA). We also determined the changes in mitochondrial membrane potential using JC-1 dye. Isolated mitochondria were incubated with 300 nM JC-1 dye (ThermoFisher, USA) for 30 min at 37 °C. Red (J-aggregates: healthy mitochondria) and green (J-monomers) fluorescence intensity were measured using a fluorescent microplate reader (BioTek, USA). Red/Green fluorescent intensity ratio was used to represent the changes in mitochondrial membrane potential. In addition, mitochondrial swelling was measured by change of mitochondrial absorbance over time, which was calculated by mitochondrial absorbance at min x/mitochondrial absorbance at min 0. The data were plotted throughout the 30 min of measurement.

#### Retrieval of PBMCs

An additional 10 ml of whole blood was collected on the day of hospital admission to retrieve PBMCs for an evaluation of mitochondrial function. PBMCs (lymphocytes and monocytes) from all patients were purified from EDTA-blood by isopycnic centrifugation using Histopaque-1077. PBMCs were stained with trypan blue dye and counted using an automated cell counter (NanoEntek, Korea). 2 × 10^5^ cells were used to determine mitochondrial function in the PBMCs.

#### Mitochondrial function analysis in PBMCs

To determine oxidative stress levels, PBMCs were stained with 2 μM DCFH-DA dye (Sigma-Aldrich, USA) for 20 min at room temperature. The fluorescence intensity of DCF was used to represent the ROS levels, and was measured by flow cytometer (FACS Celesta, BD, USA). PBMCs were also stained with 50 nM of MitoTracker Green dye (ThermoFisher, USA) for 30 min at 37 °C prior to submission to flow cytometry analysis.

#### Mitochondrial respiration analysis

PBMCs were resuspended with base medium (Agilent Technologies, USA), and centrifuged at 200 g to obtain a monolayer of the cells. Then, the cells were subjected to an extracellular flux analyzer, where a series of mitochondrial oxidative phosphorylation complex inhibitors were added as previously described^22^. Basal respiration, ATP production, maximal respiration, spared respiratory capacity, % coupling efficiency, proton leak, and non-mitochondrial respiration were analyzed automatically using the Wave program (Agilent Technologies, USA).

#### Antioxidants and mitochondrial biogenesis

Since there were some limited samples of PBMCs, 9 samples from a control, 4 samples from an early-stage ovarian cancer, and 5 samples from an advanced-stage ovarian cancer were used for analysis of protein expression. Proteins from PBMCs were extracted from RIPA buffer (Sigma-Aldrich, USA), supplemented with 1% Triton X-100 (Sigma-Aldrich, USA) and 1% protease inhibitor cocktail (Merck Millipore, USA). The proteins (1 mg/ml) were loaded onto 10% SDS–polyacrylamide gels, followed by the gel electrophoresis procedure. Then, the proteins were transferred to nitrocellulose membranes (Bio-Rad laboratories, USA) in the presence of glycine/methanol transfer buffer. The membranes were exposed to primary antibodies against superoxide dismutase 2 (SOD2) (1:1000 dilution, Cell signaling), glutathione peroxidase 4 (GPX4) (1:1000 dilution, Abcam), peroxisome proliferator-activated receptor gamma coactivator 1-alpha (PGC1α) (1:500 dilution, Santa Cruz), and Actin (1:2000 dilution, Santa Cruz) for at least 16 h. Bound antibodies were detected by horseradish peroxidase conjugated with anti-mouse/rabbit IgG. Enhanced chemiluminescence (ECL) detection reagents (Bio-Rad laboratories, USA) were employed to visualize peroxidase reaction products. Images of western blot were taken from the ChemiDoc Touching system (Bio-Rad laboratories, USA), and a densitometric analysis was performed using ImageJ program (NIH, USA)^23^.

### Statistical analysis

The comparisons between mitochondrial function profiles, including mitochondrial mass, oxidative stress, mitochondrial respiration, ROS, MMP changes, and mitochondrial swelling among the groups were made using student’s t-test and analysis of variance (ANOVA). Categorical variables were compared using chi-squared or Fisher Exact test, as appropriate. A p-value of < 0.05 was considered statistically significant.

## Results

### The characteristics of all participants

The demographic and pathological characteristics of the participants with epithelial ovarian cancers, including early-stage and advanced stage, and those in the control group are shown in Table 1. There were no statistically significant differences with regard to age, menopausal status, and histologic types among the groups. Of note, while endometrioid carcinoma and other histologic types predominated in the early-stage cancer group (approximately 70%), high-grade serous histology was found in almost half of the participants who had advanced stage cancer.

**Table 1:** Demographic data of all participants.

Demographic data (N = 57)	Control (N = 21)	Epithelial ovarian cancer patients		p-value^$^
		Early stage (N = 20)	Advanced stage (N = 16)	
Age	52 (12)	58 (12)	55 (12)	0.248
Menopause n (%)	12 (57%)	5 (25%)	6 (38%)	0.107
Cell types of tumors				0.099
High grade serous		4 (20%)	7 (44%)	
Endometrioid		6 (30%)	1 (6%)	
Clear cells		2 (10%)	4 (25%)	
Others (mucinous, mixed epithelium, etc.)		8 (40%)	4 (25%)	

Data are presented as median (IQR) or number (percentage of the control group, early stage, or advanced stage).

^$^Analysis of variance p-value.

### The profiles of mitochondrial functions in PBMCs

**Figure 2:**
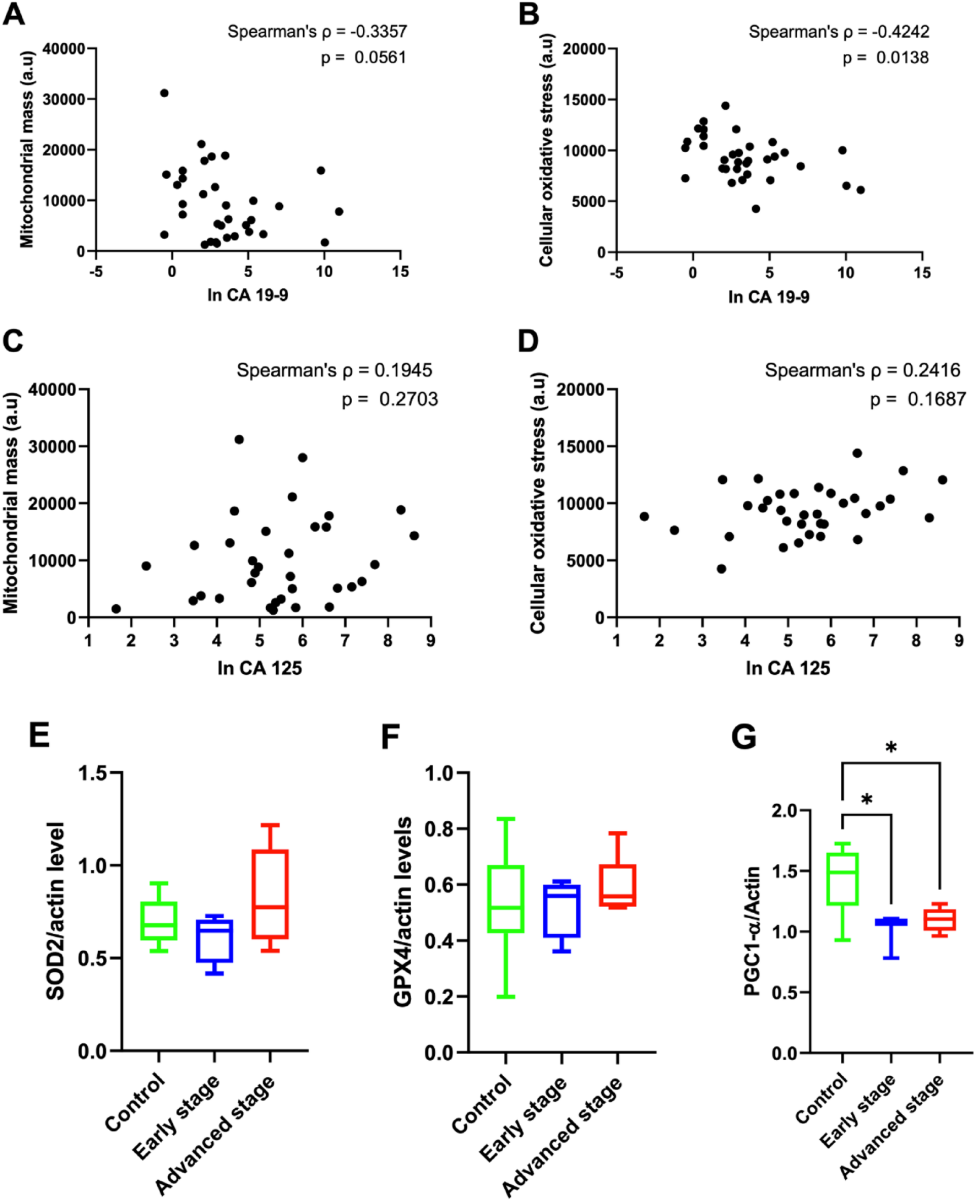
Correlation analysis between tumor markers and mitochondrial function in peripheral blood mononuclear cells (PBMCs) (**A**) ln CA 19-9 and mitochondrial mass in PBMCs, (**B**) ln CA 19-9 and cellular oxidative stress in PBMCs, (**C**) ln CA 125 and mitochondrial mass in PBMCs, (**D**) ln CA 125 and cellular oxidative stress in PBMCs, (**E**) superoxide dismutase (SOD) protein expression, (**F**) glutathione peroxidase 4 (GPX4) protein expression, and (**G**) peroxisome proliferator-activated receptor gamma coactivator 1-alpha (PGC1-α) protein expression. The comparisons in western blot analysis were conducted using the Kruskal Wallis test with uncorrected Dunn’s test. *p < 0.05 vs control.

The oxidative stress levels data are presented in Table 2 as median (IQR) values (Control = 11,079 (1879), Early stage of Epithelial Ovarian Cancer patients = 9251 (3512), and Advanced stage of Epithelial Ovarian Cancer patients = 9403 (2342)). Our findings indicate that the cellular oxidative stress was significantly lower in the PBMCs derived from participants with ovarian cancer in both early and advanced stages compared to those in the control group. In addition, the mitochondrial mass was significantly smaller in the PBMCs from participants with early-stage and advanced stage epithelial ovarian cancer, when compared to those from the control group. We investigated the impact of various histological types of epithelial ovarian cancer on mitochondrial function parameters in PBMCs. Our analysis revealed no significant difference in the cellular oxidative stress and mitochondrial mass across the different histological types of epithelial ovarian cancer (Table S1). Interestingly, there were negative associations between the level of tumor marker CA 19-9 and cellular oxidative stress (Spearman’s ρ = − 0.4242, p = 0.0138) and mitochondrial mass (Spearman’s ρ = − 0.3357, p = 0.0561) derived from PBMCs in the cancer groups (Fig. 2A,B). However, the levels of the tumor marker CA 125 did not demonstrate an association with those parameters (Fig. 2C,D). Since our data demonstrated that oxidative stress was lower in PBMCs from the participants with ovarian cancer, antioxidants, SOD2 and GPX4, were investigated as indicators of the mechanism responsible for a reduction in oxidative stress. However, SOD2 and GPX4 protein expression did not differ between the groups (Fig. 2). Additionally, a decrease in PGC1-α protein expression was observed in PBMCs from participants with early-stage and advanced stage epithelial ovarian cancer (Fig. 2G), indicating that patients with epithelial ovarian cancer exhibited a disruption in mitochondrial biogenesis, possibly leading to the reduction of mitochondrial mass in PBMCs. The full analysis of the western blot is shown in supplementary Fig. S1.

**Table 2:** Parameters of mitochondrial function in PBMCs from all participants.

Mitochondrial function parameters	Control (N = 21)	Epithelial ovarian cancer patients		p-value^$^
		Early stage (N = 20)	Advanced stage (N = 16)	
Cellular oxidative stress (a.u)	11,079 (1879)	9251 (3512)*	9403 (2342)*	**0.007**
Mitochondrial mass (a.u)	18,281 (14,790)	6954 (9704)*	10,022 (13,593)*	** < 0.001**
Mitochondrial respiration (OCR)				
Basal respiration	83.752 (61.790)	112.402 (64.180)	119.397 (91.670)	0.182
ATP production	79.665 (43.880)	94.821 (54.200)	114.109 (75.140)	0.227
Maximal respiration	210.346 (129.300)	175.213 (153.430)	199.841 (131.260)	0.768
Spared respiratory capacity	136.229 (84.130)	69.532 (104.240)	73.063 (79.560)*	**0.039**
% Coupling efficiency	90.000 (14.500)	86.992 (25.840)	83.000 (16.250)	0.151
Proton leak	9.464 (12.530)	12.199 (20.930)	15.504 (23.960)	0.186
Non-mitochondrial respiration	35.869 (25.710)	40.523 (25.670)	47.497 (49.120)	0.209

Data are presented as median (IQR).

*ATP* adenosine triphosphate, *a.u* arbitrary units, *OCR* oxygen consumption rate.

*p < 0.05 vs. control.

^$^Analysis of variance p-value.

Significance values are in bold.

### Alterations in mitochondrial ROS production, mitochondrial membrane potential changes and mitochondrial swelling in ovarian tissues/tumors

**Figure 3:**
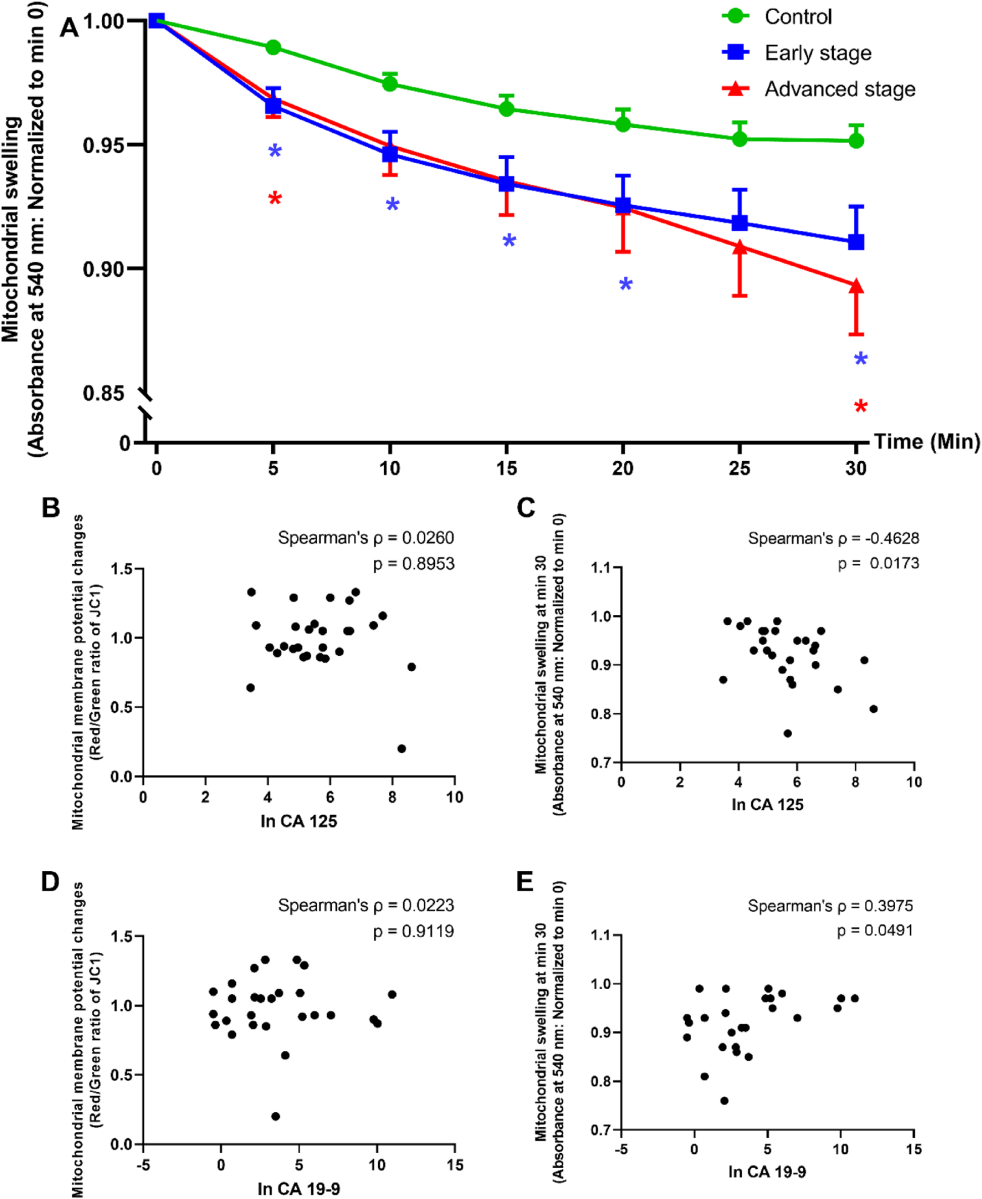
Correlation analysis between tumor markers and mitochondrial function in ovarian tissue. (**A**) Mitochondrial swelling categorized by groups (control, early stage, and advanced stage). The comparisons were conducted by two-way ANOVA with Dunnett's multiple comparisons test. *p < 0.05 vs control in the same time point. (**B**–**E**) Scatter plots between tumor markers and mitochondrial parameters in ovarian tissue. (**B**) ln CA 125 and mitochondrial membrane potential changes, (**C**) ln CA 125 and mitochondrial swelling at 30 min, (**D**) ln CA 19-9 and mitochondrial membrane potential changes, and (**E**) ln CA 19-9 and mitochondrial swelling at 30 min.

The mitochondrial ROS levels in the ovarian tissues/tumors of participants in the early-stage cancer, advanced stage cancer, and the control groups were comparable. However, mitochondrial membrane depolarization indicated by a significant decrease in the red/green fluorescence intensity ratio of JC1 was evidenced in the early-stage and advanced stage cancer groups (Table 3). Furthermore, significant mitochondrial swelling was documented in both early-stage and advanced stage cancer groups (Fig. 3A). In the participants with cancer, the absorbance ratio at 30 min, which indicates mitochondrial swelling (lower absorbance ratio representing more mitochondrial swelling), was negatively associated with the level of CA 125 (Spearman’s correlation ρ = − 0.4628, p = 0.0173; Fig. 3C), and positively associated with the level of CA 19-9 (Spearman’s correlation ρ = 0.3975, p = 0.0491; Fig. 3E). However, the level of mitochondrial membrane potential change was not associated with the levels of either tumor marker (Fig. 3B,D).

**Table 3:** Mitochondrial function parameters in ovarian tissues/tumors from all participants.

Mitochondrial function parameters	Control (N = 21)	Epithelial ovarian cancer patients		p-value^$^
		Early stage (N = 20)	Advanced stage (N = 16)	
Mitochondrial ROS level (a.u)	2540 (1331)	1962 (4681)	2732 (3028)	0.844
Mitochondrial membrane potential changes (red/green ratio of JC1)	1.760 (0.540)	1.065 (0.360)*	0.934 (0.190)*	** < 0.001**

Data are presented as median (IQR).

*ROS* reactive oxygen species.

*p < 0.05 vs. control.

^$^Analysis of variance p-value.

Significance values are in bold.

## Discussion

We found that the participants with epithelial ovarian cancer had significantly lower levels of cellular oxidative stress in the PBMCs compared to control. In addition, we noted a significantly smaller mitochondrial mass in PBMCs of the cancer groups. The mitochondrial respiratory parameters measured from the PBMCs were similar among the early-stage cancer, the advanced stage cancer, and the control groups. From the assessment of the ovarian tissues/tumors, while the mitochondrial ROS levels were not significantly different between the cancer and the control group, the mitochondrial membrane depolarization was evident in both the early-stage cancer and advanced-stage cancer groups. Furthermore, significant mitochondrial swelling was discovered in the early- and advanced-stage cancer participants.

Intracellular ROS, mostly derived from superoxide (O_2_^−^), is generated by one-electron reduction of O_2_ from the mitochondrial electron transport chain and released into the mitochondrial matrix and intermembrane space. Subsequently, it is converted to hydrogen peroxide (H_2_O_2_) by SODs either in the matrix by SOD2 and further diffused to the cytosol or in the cytosol by SOD1. The cytosolic H_2_O_2_ is the major form of intracellular signaling of ROS. This peroxide has an important role in cellular adaptation to an unfavorable environment, including hypoxia and starvation, and also in the regulation of autophagy, differentiation, and immunity. At the physiologic baseline, the tonal level of ROS is essential to the maintenance of cellular homeostasis. During environmental stress, the increase and fluctuating levels of ROS send signals to various molecular targets, currently still unclear, to initiate many cellular adaptations^24^. In the hypoxic conditions invariably associated with carcinogenesis, the increased ROS initiates and enhances the cellular responses through regulation of hypoxia inducible factors (HIFs) to increase oxygen supply and decrease oxygen demand by facilitating erythropoietin expression to boost red blood cell production, vascular endothelial growth factor to promote neovascularization, and glycolytic enzymes to sustain ATP production and diminish ATP consumption. ROS may regulate intracellular resources for survival during nutrient starvation by inducing autophagy^24^. In the course of carcinogenesis, in particular, the elevation of ROS plays a major role in promoting cell proliferation, cell survival, angiogenic growth factor, cell apoptosis, cancer metastasis, and inflammatory response^6,25^. Despite these essential functions regulated by ROS, a sustained increase in ROS production to the level that surpasses antioxidant defenses would create oxidative stress resulting in damage to proteins, lipids, and nucleic acids, resulting in cell death. Therefore, cells need certain mechanisms to limit ROS production under prolonged hypoxic conditions to avoid cellular damage and death^6,24,26–28^. Our study demonstrated a lower level of oxidative stress in the PBMCs from the cancer group, both early- and advanced stages, compared to the control group. However, the mitochondrial ROS levels in ovarian tumor tissues were not significantly different from those in normal ovarian tissues. These findings suggest a potential existence of certain mechanisms of epithelial ovarian cancer that prevent ROS overproduction and promote cancer cell survival. Interestingly, the significantly smaller mitochondrial mass associated with both early- and advanced stages of cancer suggests that reducing mitochondria quantity through autophagy of mitochondria (mitophagy) may play a major regulatory role in this circumstance. In the ROS-mitophagy feedback loop, the increased ROS production provokes mitophagy, which further restricts ROS production by reducing mitochondrial mass^24^. Our findings may seem in contrast with a recent study examining mitochondrial biogenesis in 16 human serous and mucinous ovarian cancer tissue samples compared to 18 controls^29^. In that study, the authors documented increased mitochondrial biogenesis in ovarian cancer from an increase in the number of mitochondria along with increased PGC1α level, mitochondrial transcription factor (TFAM) level, and mitochondrial DNA (mtDNA) content. We believe that the conflicting findings could represent the dynamic nature of mitochondrial functions in the process of carcinogenesis. In the early phase, when mitochondrial ROS production is increasing in response to hypoxia or starvation associated with cancer development, biogenesis may increase resulting in increased mitochondrial mass. Subsequently, when the ROS level reaches the threshold of oxidative stress, mitophagy may commence regulatory action by impeding biogenesis leading to the decrease in mitochondrial mass as observed in our study. The literature supports the idea that genetic alterations can trigger metabolic changes that promote cancer progression. Our results not only support this notion but also highlight the possible regulatory role of metabolic alteration through the ROS-mitophagy feedback loop as part of the entire carcinogenesis process. When our findings are combined with the existing literature, they further underscore the dynamic nature of mitochondrial functions in the process of carcinogenesis.

However, with the cross-sectional data available from fresh human PBMCs and ovarian tissues, it is not possible to illustrate the entire picture of the highly dynamic process of change in mitochondrial function during carcinogenesis. Nevertheless, we propose that the findings of decreased mitochondrial mass and the low level of cellular oxidative stress could signify the mitochondrial function changes associated with fully developed ovarian cancers. We investigated the impact of various histological types of epithelial ovarian cancer on mitochondrial function parameters in PBMCs. Our analysis revealed no significant difference in the cellular oxidative stress and mitochondrial mass across the different histological types of epithelial ovarian cancer (as shown in the supplementary Table S1). However, we acknowledge that the statistical power to detect minor differences is limited due to the small number of samples in each histological category. We also noted the generally comparable mitochondrial respiratory functions between the cancer and the control groups regardless of the cancer histology.

A proton gradient between the intermembrane space (IMS) and mitochondrial matrix is generated by pumping protons from the matrix to the IMS at complexes I, III, and IV of mitochondrial electron transport chain located at the inner mitochondrial membrane (IMM). This process leads to a higher proton concentration in the IMS than in the matrix, creating MMP, which is essential for normal functioning of ATP synthase and ATP synthesis. It has been proposed that the MMP of cancer cells in general is higher (hyperpolarized) than those in normal cells (approximately − 220 mV vs. − 108 to − 180 mV) due to dysfunctional ATP synthase causing accumulation of protons in the IMS^26,30^. However; this was not the case in our study. We found that the MMP was lower (depolarized) in both early- and advanced stage cancer compared to control without decreased ATP production.

In summary, in this study we demonstrated the dynamic nature of mitochondrial ROS production, biogenesis, and respiratory function in response to epithelial ovarian carcinogenesis. The flexibility of mitochondrial functions under diverse conditions makes it a challenging therapeutic target. It is important to note that our study only provides cross-sectional data on fresh human PBMCs and ovarian tissues, and therefore, cannot provide a complete picture of the highly dynamic process of changes in mitochondrial function during epithelial ovarian carcinogenesis. In addition, our results were not sufficient to draw any meaningful conclusion to support our hypothesis regarding the correlation between the specified mitochondrial functions from PBMCs, ovarian tissues, and tumor biomarkers. Furthermore, we acknowledge that the statistical power to detect minor differences in the outcomes of this study is limited due to the small number of samples, especially in each histological category of epithelial ovarian cancer. Future studies with larger sample size evaluating a comprehensive profile of mitochondrial function in both PBMCs and ovarian tissues is worthwhile.

### Supplementary Information

Supplementary Figure S1.

Supplementary Table S1.

## Data Availability

Data will be made available on request to the corresponding author.
