# Supplementary figures and images for: Alterations in mitochondria isolated from peripheral blood mononuclear cells and tumors of patients with epithelial ovarian cancers
Source: Sci Rep. 2024 Jan 2;14:15. doi: 10.1038/s41598-023-51009-z (PMC10762226; doi:10.1038/s41598-023-51009-z)

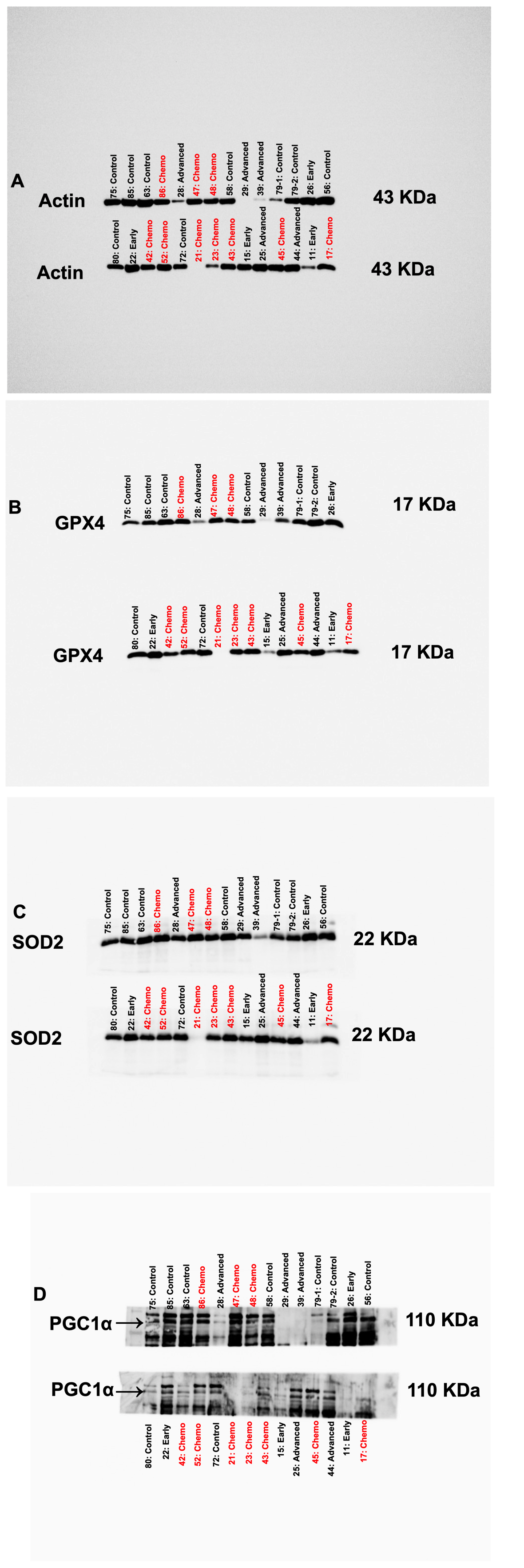

Supplement: Supplementary file 1 — Supplementary Figure S1. [file 41598_2023_51009_MOESM1_ESM.tiff]
