# Supplementary material for: Alterations in mitochondria isolated from peripheral blood mononuclear cells and tumors of patients with epithelial ovarian cancers
Source: Sci Rep. 2024 Jan 2;14:15. doi: 10.1038/s41598-023-51009-z (PMC10762226; doi:10.1038/s41598-023-51009-z)
Supplement: Supplementary file 2 — Supplementary Table S1. [file 41598_2023_51009_MOESM2_ESM.docx]

**Table S1** Comparison of mitochondrial function parameters in PBMCs among different histological types of epithelial ovarian cancer patients

| **Mitochondrial function parameters** | **Endometrioid**  **(N = 7)** | **Clear cell**  **(N = 6)** | **High-grade serous**  **(N = 11)** | **p-value^$^** |
| --- | --- | --- | --- | --- |
| Cellular oxidative stress (a.u) | 11034 (4633) | 8920 (4000) | 8638 (2406) | 0.401 |
| Mitochondrial mass (a.u) | 11150 (12154) | 9362 (18790) | 7652 (11769) | 0.997 |
| ***Mitochondrial respiration (OCR)*** |  |  |  |  |
| Basal respiration | 79.973 (96.600) | 134.206 (174.060) | 87.321 (82.880) | 0.153 |
| ATP production | 99.000 (98.720) | 118.792 (112.840) | 80.128 (77.060) | 0.410 |
| Maximal respiration | 152.252 (310.960) | 278.332 (246.52) | 171.606 (129.440) | 0.173 |
| Spared respiratory capacity | 55.266 (223.540) | 109.734 (148.880) | 64.869 (63.460) | 0.805 |
| **%Coupling efficiency** | 90.000 (41.030) | 69.833 (22.250) | **89.244 (35.750)*** | **0.011** |
| Proton leak | 5.733 (13.120) | 25.242 (71.920) | 7.193 (13.190) | 0.116 |
| Non-mitochondrial respiration | 43.792 (56.770) | 46.717 (50.660) | 57.114 (86.880) | 0.755 |

Data were presented as median (IQR). *p<0.05 vs. clear cell; ^$^Analysis of variance p-value

**Abbreviation:** ATP: adenosine triphosphate; a.u: arbitrary unit; OCR: oxygen consumption rate.
